# Relationship between non-typhoidal *Salmonella* dose and food poisoning in humans: A systematic review

**DOI:** 10.3934/microbiol.2025014

**Published:** 2025-04-14

**Authors:** Ophélie Colin, Laure David, Jean-Denis Bailly, Pedro Henrique Imazaki

**Affiliations:** 1 Educational Unit on Food Hygiene and Industry, Université de Toulouse, ENVT, Toulouse, France; 2 IRSD, Université de Toulouse, INSERM, INRAE, ENVT, UPS, Toulouse, France; 3 LCA, Université de Toulouse, INRAE, INPT, ENVT, Toulouse, France; 4 INTHERES, Université de Toulouse, INRAE, ENVT, Toulouse, France

**Keywords:** Dose-response relationship, epidemiology, food safety, foodborne illness, foodborne outbreaks, gastroenteritis, infectious dose, mathematical modelling, non-typhoidal *Salmonella*, public health, risk assessment, systematic review, zoonotic pathogens

## Abstract

Food safety is a major public health concern. The zoonotic pathogen non-typhoidal *Salmonella*, responsible for salmonellosis, is a leading cause of bacterial food poisoning globally, making its detection and control essential. Understanding the infectious dose of *Salmonella* is crucial for identifying appropriate risk management strategies; however, significant uncertainties remain, warranting a systematic review. Following PRISMA guidelines, we conducted a comprehensive search across multiple databases (Web of Science, PubMed, and CAB Abstracts) to identify relevant studies examining the relationship between *Salmonella* dose and foodborne illness in humans. Four main types of studies were identified: experimental trials, case reports, case series, and mathematical modelling. An analysis of these studies revealed their respective strengths and limitations. The data showed considerable variability, with the dose required to cause illness depending on factors such as *Salmonella* serovar, food type, and the health status of the exposed population. A key challenge identified was the lack of sufficient data on collective food poisoning incidents, which complicates the development of more reliable dose-response models. Despite these limitations, this review underscores the importance of targeted food safety interventions and risk assessments tailored to specific food products and population groups. The findings provide a foundation for enhanced food safety measures and support ongoing efforts to protect public health from foodborne illnesses.

## 1. Introduction

Food safety remains a critical public health concern worldwide, and the urgency of addressing this issue cannot be overstated. Non-typhoidal *Salmonella*, including serovars Enteritidis and Typhimurium, is one of the leading causes of bacterial food poisoning, responsible for a significant number of salmonellosis cases each year [1]. Salmonellosis is a zoonotic disease that typically causes acute enterocolitis, characterised by symptoms such as diarrhoea (sometimes bloody), abdominal cramps, fever, nausea, and vomiting, which are mild and self-limiting in most individuals. The illness can also manifest as an invasive febrile disease, with bacteraemia, meningitis, or focal infections that, if left untreated or inadequately managed, can be fatal [2],[3]. Transmission primarily occurs through the consumption of contaminated food, particularly—but not exclusively—products of animal origin [4]. *Salmonella*'s ability to survive in various environmental conditions, including different temperatures, pH levels, and moisture content, makes it a persistent and formidable threat to food safety [5]. Despite extensive measures to control and prevent its transmission to humans, outbreaks continue to occur, highlighting the need for a deeper understanding of these infections, particularly the infectious dose, to enhance strategies for managing this pathogen.

The infectious dose—the minimum number of bacteria required to cause illness—of *Salmonella* can vary considerably depending on several factors. These include the specific serovar of *Salmonella*, each exhibiting different levels of virulence [6], the type of food in which *Salmonella* is present [7], and the susceptibility of the exposed population [3]. For example, high-fat and high-protein foods can protect *Salmonella* against gastric acidity, potentially lowering the number of bacteria required to cause infection. This protective effect occurs because fats and proteins buffer the acidic environment of the stomach, allowing more bacteria to survive and reach the intestines, where infection occurs [8]. Moreover, vulnerable populations such as the elderly, young children, and immunocompromised individuals are especially at risk of severe illness from even lower doses of *Salmonella* [3]. For instance, neonates and very young infants (under five years) have less developed immune systems than healthy adults, making them more vulnerable to infections. Similarly, elderly individuals (65 years and older) often experience weakened immune responses due to age-related changes, and those with immunocompromising conditions such as HIV/AIDS or those undergoing chemotherapy are less capable of fighting infections [9].

Previous studies have reported a wide range of infectious doses for different *Salmonella* serovars. Some studies suggest that as few as 10–100 bacteria can cause illness, while others indicate doses as high as 10,000,000,000 [10]. Given the significant discrepancies in infectious doses reported across the literature, there is a pressing need for a systematic review to synthesise existing knowledge and identify gaps in our understanding.

In this context, this review aims to provide an objective and reproducible synthesis of the relationship between non-typhoidal *Salmonella* doses and foodborne illness in humans. The primary research question addressed is: “What is the dose-response relationship between the dose of non-typhoidal *Salmonella* and foodborne illness in humans?” A secondary question explored is: “Which factors influence this dose-response relationship?”

By analysing data from a range of studies, including experimental trials, case reports, case series, and mathematical modelling, this review seeks to deepen our understanding of the dose-response relationship, a critical step in the development of effective risk assessments. More accurate risk assessments can subsequently inform improved control measures and policies as well as guide targeted interventions aimed at reducing the incidence of salmonellosis. Ultimately, this review supports ongoing efforts to protect public health from *Salmonella* contamination in food products, mitigate the impact of this pathogen, and identify the missing data necessary to achieve a comprehensive understanding of the associated risks.

## 2. Materials and methods

The literature search for this systematic review was conducted following the Preferred Reporting Items for Systematic Reviews and Meta-Analyses (PRISMA) guidelines [11]. All steps of the systematic review were carried out using the free web tool CADIMA [12].

### 2.1. Modified PICO elements

- Population: humans
- Intervention: exposure to non-typhoidal *Salmonella* in food
- Comparator: dose-effect
- Outcome: salmonellosis

### 2.2. Search strategy

For this systematic review, relevant documents were collected using peer-reviewed databases, including Web of Science, PubMed, and CAB Abstracts. The keywords and search strings applied to these electronic databases included: *Salmonella* AND (“Foodborne” OR “Food poisoning”) AND Dose. The search was conducted between June and July 2022. To ensure the robustness of our search strategy, a quality control process was performed by selecting a random sample of 10 relevant articles identified through manual searches. These articles were cross-checked against the results obtained from the database searches to verify that the search string effectively captured all relevant studies. Any discrepancies between the manually identified articles and the search results were analysed, and adjustments to the search strategy were made accordingly.

### 2.3. Selection process

The selection process involved a two-stage screening to compile a list of documents pertinent to our research question. O.C. and P.H.I. independently conducted blinded screenings to ensure objectivity.

First, inclusion and exclusion criteria were defined for the title and abstract review of the identified records (search results from bibliographic databases), allowing for an initial filtering of the literature. According to the PI(E)CO principles, the inclusion criteria were as follows:

- Population: humans
- Exposure: *Salmonella* transmitted by food or water
- Outcome: non-typhoidal salmonellosis

The exclusion criteria were:

- Records focusing on animal populations without any reference to human subjects
- Records where *Salmonella* was transmitted through means other than food or water
- Records concerning typhoidal *Salmonella*

If the necessary information in a record was not clearly stated in the title or abstract, it was selected for the next screening stage.

In the second stage, full-text screening was performed for reports (full-text documents) that passed the initial filter using additional criteria:

- The full text was accessible
- The text was written in one of the following languages: English, Spanish, Portuguese, French, German, Italian, or Dutch
- The document reported an infectious dose or a dose-response relationship, along with a description of the methodology used to obtain this data

In cases of discrepancies between the two reviewers' results in either screening phase, discussions were held to reach a consensus on the inclusion or exclusion of problematic documents. Following these screenings and harmonisation, a definitive list of documents for data extraction was compiled. The reference lists of the included documents were also reviewed to identify and add relevant records that may have been missed in the original search. Multiple studies (investigations) could be identified within a single document, and these were treated individually during the data extraction phase.

### 2.4. Data extraction

A data extraction grid was established to facilitate a systematic and efficient analysis of the studies by extracting relevant information. This grid was applied to the selected studies using the CADIMA tool. The categories and labels, designed to be as comprehensive as possible, are presented in Table 1.

**Table 1.** Data extraction categories, label, and information to be collected.

Category	Label	Information to be collected
General information	Article ID	Assigned by CADIMA
	Study ID	Assigned by CADIMA
	Authors	Names of the authors
	Year of publication	The year the study was published
	Title	Title of the article
Study characteristics	Study name	Name of the study
	Type of study	Case report, case series, randomised controlled trial, or mathematical modelling
	Type of results	Infectious dose, dose-response relationship, or mathematical modelling
	WHO region	World region as defined by WHO
	Country	Country where the study was conducted
	Study period	Duration over which the study was conducted
Population details	Study population	General population, children, elderly, volunteers, etc.
	Number of participants	Size of the population in the study
	Number of exposed individuals	Number of individuals exposed to *Salmonella*
	Number of ill individuals	Number of individuals who developed illness
	Population information	Gender, age, health status, location, etc.
Exposure details	Mode of transmission	Individual cases of foodborne illness, foodborne disease outbreaks, etc.
	Contaminated food	Name of the implicated food
	Level of contamination	Level of contamination in the food
	Amount of food ingested	Quantity of contaminated food consumed
	Dose ingested	The actual dose of *Salmonella* ingested
	Type of dose obtained	CFU/g, MPN/g, ID50, etc.
Laboratory methods	*Salmonella* serovar	Serovar of *Salmonella* identified
	Laboratory identification method	Methods used to identify *Salmonella*
	Quantitative laboratory method	Methods used to quantify *Salmonella*
	Additional information	Conditions of sample storage, time between contamination and analysis, etc.
Clinical outcomes	Salmonellosis definition	Symptoms sought by authors and/or presented by patients
	Morbidity	Attack rate
	Incubation period	Time from exposure to onset of illness
	Duration of symptoms	Length of time symptoms persisted
Main findings and conclusions	Main findings	Key results of the article
	Main conclusions	Conclusions drawn by the authors
	Additional notes	Any other relevant information

### 2.5. Risk of bias assessment

We established a set of criteria to identify potential sources of bias, which enabled us to assess the rigour of each study. These criteria, outlined in Table 2, were rated on scales of one to two or three. Higher scores indicate that the study exhibited minimal bias, employed a robust methodology, and demonstrated careful consideration of potential limitations.

**Table 2.** Criteria used in the assessment of bias risk of selected articles.

Criteria	Source of bias	Scale applied
Study using data from its own work or another source	Source of data	1-External; 2-Mixed; 3-Internal
Data used clearly stated (with source if needed)	Availability of data	1-No; 2-Unclear; 3-Yes
Concentration and ingested quantity expressed	Expression of dose	1-Unclear; 2-Estimated; 3-Determined
Storage conditions of samples considered in dose calculations	Storage conditions of samples	1-Not mentioned; 2-Discussed; 3-Discussed and used in calculations
Estimation or measurement of the size of the exposed population	Size of exposed population	1-Unclear; 2-Estimated; 3-Measured
Estimation or measurement of the size of the ill population	Size of ill population	1-Unclear; 2-Estimated; 3-Measured
Characteristics of individuals clearly presented	Health status of the population	1-Not described; 2-Described
Appreciation of population size with critical evaluation by the author	Population size	1-No; 2-Yes
Possibility to calculate an attack rate	Attack rate	1-No; 2-Yes
Distinction made between infection and disease	Infection or disease	1-No; 2-Yes
Contaminated food clearly identified	Contaminated food	1-No; 2-Unclear; 3-Yes
Composition or characteristics of the contaminated food stated	Characteristics of the food	1-No; 2-Unclear; 3-Yes
Critical evaluation by the authors of the study results and comparison with other studies	Discussion of results	1-No critical evaluation by authors; 2-Critical evaluation by authors
Critical evaluation by the authors of factors that may influence their results	Discussion of factors influencing results	1-No critical evaluation by authors; 2-Critical evaluation by authors

## 3. Results

### 3.1. Literature search and selection process

Following the literature search described previously, 982 records (bibliographic descriptions of reports) were identified across the three databases, and 367 duplicates were removed. An initial screening of the remaining 615 records was conducted by reviewing the titles and abstracts based on the predefined criteria, leading to the exclusion of 517 records. Consequently, 98 reports (full-text documents) were deemed eligible for retrieval, but 13 were unavailable. A full-text screening of the remaining 85 reports was performed, resulting in the retention of only 12. Most of the 73 excluded reports did not meet the criteria of providing data on an infectious dose or a dose-response relationship, along with a description of the methodology used to obtain these data.

**Figure 1.**
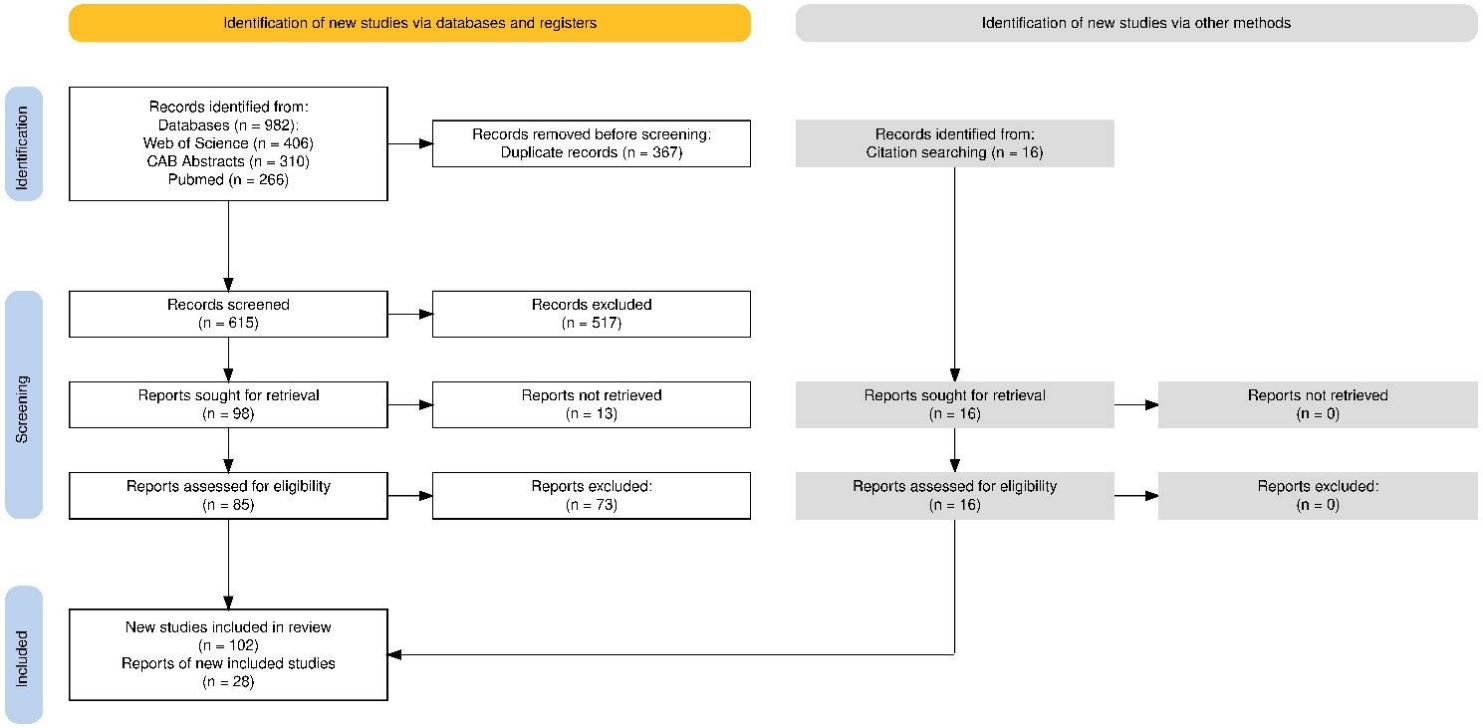
Summary of the selection of studies included in the review according to PRISMA guidelines.

Additional searches through the bibliographic references of these articles led to the inclusion of 16 new records in the data extraction phase. In total, our review included 28 reports, which collectively described 102 studies. Since a single article may report multiple studies—such as investigations of several outbreaks or case series—there is a distinction between the number of manuscripts and the number of studies included in the review. Each study was treated individually during data extraction. Figure 1, a PRISMA flow diagram [13], illustrates the results of the screening process.

Each included study underwent a risk of bias assessment, allowing for the identification of potential biases that could affect the reliability of the findings. No study exhibited a risk of bias significant enough to warrant exclusion from this review. The strengths and limitations of each article are addressed in Section 4.

### 3.2. Characteristics of selected studies

This review included seven case reports (6.9%), 71 case series (69.6%), 13 experimental studies (12.7%), and 11 mathematical models (10.8%). The seven case reports documented sporadic incidents where individuals with unique characteristics became ill after consuming a specific dose of *Salmonella*. The 71 case series involved detailed descriptions of multiple clinical cases following exposure to the same hazard source, specifically the consumption of food contaminated with *Salmonella*. The 13 experimental studies were randomised controlled trials conducted by McCullough & Eisele between 1950 and 1951 [14]–[16]. Finally, the 11 studies using mathematical modelling aimed to establish a dose-response relationship and estimate an infectious dose. These models relied on data from other studies, including those by McCullough & Eisele [14]–[16], as well as foodborne disease outbreaks, combined with mathematical tools to address the lack of field data.

The temporal distribution of different types of studies from 1950 to the present shows a distinct pattern. Clinical trials on humans, the oldest type of study retrieved, have not been conducted since the 1950s due to obvious ethical concerns. Case reports and case series have been used over time to determine an infectious dose in real-world conditions. The use of mathematical models to determine dose-response relationships began in the late 1990s and remains a prevalent method, often relying on data from documented salmonellosis outbreaks.

The geographic distribution of data sources is another relevant parameter, as the distribution of *Salmonella* serovars and strains is not uniform worldwide [17]. Therefore, the conclusions of these studies may not always be applicable to strains found in different regions or countries. Additionally, methods for combating *Salmonella* vary by region, affecting both hazard and risk levels. The Americas, South-East Asia, and Europe accounted for 47.1%, 40.2%, and 6.9% of the studies, respectively. No studies from the African, Eastern Mediterranean, or Western Pacific regions were included in this review. Studies collecting data from multiple regions accounted for 5.9%.

The characteristics of the selected studies are summarised in Table 3. The table highlights the frequencies of case reports, case series, randomised controlled trials, and mathematical models, as well as the regions from which these studies originated, across three publication periods: 1951–1974, 1975–1999, and 2000–2019.

**Table 3.** Type of studies and regions by year of study publication.

	Year of publication		
	1951–1974	1975–1999	2000–2019
Type of study			
Case reports	-	7 (6.9%)	-
Case series	14 (13.7%)	16 (15.7%)	41 (40.2%)
Randomised controlled trials	13 (12.7%)	-	-
Mathematical models	-	2 (2.0%)	9 (8.8%)
Region			
Americas	27 (26.5%)	17 (16.7%)	4 (3.9%)
South-East Asia	-	-	41 (40.2%)
Europe	-	7 (6.9%)	-
Multiple regions	-	1 (1.0%)	5 (4.9%)

A summary of each selected study is presented in Section 3.3. For narrative purposes, the studies are grouped according to their type: case reports, case series, experimental studies, and mathematical models. This structure allows for a clearer comparison of the findings across different study designs.

### 3.3. Findings from case reports, case series, experimental studies, and mathematical models

#### 3.3.1. Case reports

A case report is a detailed presentation of a single patient, including their clinical course, diagnosis, treatment, and outcomes. In this review, the case reports illustrate individual responses to doses of *Salmonella* transmitted via food. D'Aoust studied a large foodborne outbreak of salmonellosis in Canada, involving more than 1500 cases of illness between December 1983 and July 1984. Cheddar cheese, manufactured by a plant in eastern Canada and found to contain *Salmonella* Typhimurium PT10, was identified as the primary vehicle of infection. However, epidemiological investigations indicated only six separate incidents in which cheese samples were recovered to determine the infectious dose [18]. Three of these incidents involved only one person each and are summarised in this section. The three other incidents, which involved more than one person, are presented in the next section alongside other case series.

Similarly, Vought & Tatini investigated a large multistate outbreak of foodborne illness in the United States, attributed to *Salmonella* Enteritidis contamination of ice cream products manufactured between late August and September 1994. They identified six incidents where the potential infectious dose of *S*. Enteritidis in ice cream could be estimated [19]. Two of these incidents are summarised in this section, while the three case series are treated in the next section.

Finally, two letters from George and Lipson, published by *The Lancet* in 1976, provided evidence of small infectious doses of *Salmonella*. They reported cases of salmonellosis in hospitalised children treated with pancreatic extract contaminated with *Salmonella* Schwarzengrund [20],[21]. Although pancreatic extracts cannot be classified as food, these two studies were included due to the originality of this source of *Salmonella*.

Regarding individual characteristics, three of the seven cases described involved children [19]–[21], two of whom were hospitalised at the time of contamination [20],[21]. Two cases involved adults over the age of 65 [18]. The serovars (and strains) responsible for contamination in these case reports were *Salmonella* Enteritidis PT8 [19], *S*. Typhimurium PT10 [18] and *S*. Schwarzengrund [20],[21]. Only two food matrices were represented: ice cream [19] and cheddar cheese [18]. The lowest dose recorded in the selected studies was 0.7 MPN [18] and, in all studies, the infectious doses were below 200 organisms.

A summary of selected results from the data extraction for the case reports is presented in Table 4.

**Table 4.** Summary of findings of case reports.

Exposed person and age	*Salmonella* serovar (and strain)	Contaminated food	Ingested dose^1^	Source
Female, 71 y	Typhimurium PT10	Cheddar cheese	0.7 MPN	[18]
Male, 56 y	Typhimurium PT10	Cheddar cheese	4.2 MPN	[18]
Male, 66 y	Typhimurium PT10	Cheddar cheese	6.1 MPN	[18]
Male, 45 y	Enteritidis PT8	Ice cream	18–74 MPN	[19]
Child, 8 y	Enteritidis PT8	Ice cream	6–25 MPN	[19]
Child (hospitalised), 1 y	Schwarzengrund	Pancreatic extract	< 200 CFU	[20]
Child (hospitalised), 9 mth	Schwarzengrund	Pancreatic extract	< 44 CFU	[21]

^1^ Quantity of *Salmonella* ingested, calculated as the product of the concentration of *Salmonella* in the food and the amount of food consumed.

#### 3.3.2. Case series

A case series study is an observational research method that involves the detailed reporting and analysis of a series of individuals who have a particular disease, condition, or exposure. In this review, we analysed case series documenting the transmission of *Salmonella* through food, leading to salmonellosis. Case series studies were the most prevalent in our literature search, accounting for 15/28 reports and 71/102 studies. The earliest case series addressing the infectious dose of *Salmonella* was published by Armstrong et al. in 1970. The authors investigated 14 separate banquets followed by febrile gastroenteritis due to the ingestion of imitation ice cream desserts contaminated with *Salmonella* Typhimurium and two other *Salmonella* serovars, produced by a company in New York City. Contamination resulted from the use of *Salmonella*-infected unpasteurised egg yolks that were not cooked during the ice cream manufacturing process [22].

D'Aoust et al. and Craven et al. reported an international outbreak in 1973–1974 across seven provinces in Canada and 23 states in the United States, involving chocolate balls contaminated with *Salmonella* Eastbourne, manufactured by a Canadian company. Bacteriological testing implicated cocoa beans as the probable source of *Salmonella* [23],[24]. Then, an interstate common-source outbreak of salmonellosis was detected in the United States in September and October 1975, when a ten-fold increase in *Salmonella* Newport isolates was noted through routine health surveillance. *Salmonella* Newport with the same antibiogram and phage lysis pattern as the human epidemic isolates was cultured from frozen beef patties recovered in Colorado and Florida. The contaminated beef patties originated from the same processing plant in Dallas, Texas. Although the source of the epidemic strain was not identified, it likely entered the plant before delivery [25].

In 1976, ongoing *Salmonella* surveillance in Colorado detected an epidemic of *Salmonella* Heidelberg. Epidemiological investigations linked the outbreak to cheddar cheese from a single shipment by one manufacturer. This outbreak involved milk of poor microbiological quality, an incorrectly placed filter in the pasteurisation system, and a lack of bacteriological culture or phosphatase testing of the final product, as described by Fontaine et al. [26].

In 1982, Gill et al. and Greenwood & Hooper investigated an outbreak of *Salmonella* Napoli infection in England and Wales, detected through the routine surveillance of *Salmonella* infections from hospitals and public health facilities. The investigation quickly identified two types of small chocolate-covered bars imported from Italy as the vehicle of infection; both were subsequently found to be contaminated with the organism [27],[28]. Furthermore, Taylor et al. described an outbreak of salmonellosis associated with a particularly large dose and severe illness, resulting in the death of a healthy child. In this case, *Salmonella* gastroenteritis occurred on a Wyoming farm in June 1982, where all eight individuals became severely ill after eating homemade ice cream. *Salmonella* Typhimurium was isolated from all eight patients, the remaining ice cream, and the family's hens, whose eggs were used to make the ice cream [29].

Between December 1983 and July 1984, a cheddar cheese contamination, described in the previous section, resulted in salmonellosis in nine people across three separate incidents, as detailed by D'Aoust [18]. Moreover, Kapperud et al. described an outbreak of *S*. Typhimurium infection in Norway and Finland in 1987, caused by contaminated chocolate produced by a Norwegian company. The authors suggested that the outbreak strain may have been introduced through contaminated raw ingredients imported from another country or possibly derived from an avian wildlife reservoir in Norway, with birds potentially introducing contamination somewhere along the production line [30].

Morgan et al. described a family outbreak of *Salmonella* Enteritidis PT4, in which homemade ice cream was identified as the vehicle of infection. The ice cream was likely contaminated by an infected eggshell [31]. Additionally, a large outbreak in Germany between April and September 1993 was investigated by Lehmacher et al. In this case, millions of contaminated paprika-powdered potato chip packs were distributed nationwide. A distinctive aspect of the outbreak was the involvement of multiple serovars. *Salmonella* Saintpaul, *Salmonella* Rubislaw, and *Salmonella* Javiana were all isolated within the same timeframe from paprika powder, spice mixtures, snacks, and patients [32].

Vought & Tatini's study on the multistate outbreak linked to *S*. Enteritidis contamination of ice cream, described earlier, identified three case series in which the potential infectious dose of *Salmonella* could be estimated [19]. Finally, two records contributed the most to the case series included in this review. Research by Kasuga et al. and Hara-Kudo & Takatori provided data from numerous foodborne outbreaks in Japan, summarising detailed information obtained from administrative foodborne outbreak reports provided by local governments [10],[33].

The ingested dose does not represent an infectious dose but indicates whether the amount of *Salmonella* can cause illness in part of the population. Doses causing illness in the case series varied widely, from as few as six bacteria to billions of organisms. Low ingested doses were reported in incidents involving high-fat foods, such as chocolate and cheese [23],[26],[27],[30], or in the German outbreak involving paprika-powdered potato chips, where low-contaminated mass-produced food reached millions of consumers within a short period, leading to significant public health issues [32]. In contrast, high ingested doses were associated with homemade dishes prepared with raw eggs [29],[31] and the consumption of unusually large quantities of contaminated food [29]. About one-third of the studies reported doses below 1000 organisms, one-third reported doses between 1000 and 50,000, and one-third reported doses above 50,000 organisms.

In the previously mentioned studies, the size of the populations examined varied significantly. The number of exposed individuals ranged from small family units to large-scale commercial products affecting millions. For example, D'Aoust and Vought & Tatini reported case series with only two exposed individuals [18],[19], whereas Lehmacher et al. estimated exposure to 10 million individuals [32]. In many studies, the exact number of exposed individuals is approximated, particularly in large-scale incidents. About 20% of the case series contained approximations for both exposed and ill individuals, complicating accurate assessment of the full impact of some exposures.

Various serovars were implicated in non-typhoidal salmonellosis in humans, with some serovars more commonly reported than others. For instance, *S*. Enteritidis was found in 53% of the case series and *S*. Typhimurium was present in 21%. Other serovars, such as Agona, Bareilly, Braenderup, Cerro, Eastbourne, Heidelberg, Javiana, Montevideo, Napoli, Newport, Oranienburg, Saint Paul, and Rubislaw were identified and studied in one or two outbreaks described in the selected studies.

The food matrices involved in the case series were highly variable. Often, a dish was identified without pinpointing a specific component containing the bacteria. Frequently recurring ingredients in the dishes included eggs, present in at least 16 out of 73 identified dishes (21%), chocolate in 11% of cases, cream in 9.5%, and both soy and rice, each appearing in 5% of the dishes. Unexpected items, such as paprika chips, were also implicated, demonstrating the diversity of potentially contaminated foods. Due to this variety, it is difficult to identify a single food item, apart from eggs, that is more likely to cause salmonellosis.

Relevant results from the case series studies are summarised in Table 5.

**Table 5.** Summary of findings of case series.

Population (number of sick/exposed individuals)	Salmonella serovar (and strain)	Contaminated food	Ingested dose^1^	Source
38/87 (44%)	NA^2^	Non-dairy imitation ice cream	11,300 CFU	[22]
NA^2^/100	Typhimurium var. Copenhagen	Non-dairy imitation ice cream	11,300 CFU	[22]
770/1400 (55%)	Typhimurium	Non-dairy imitation ice cream	11,300 CFU	[22]
57/80 (71%)	Typhimurium and Braenderup	Non-dairy imitation ice cream	11,300 CFU	[22]
129/202 (64%)	Typhimurium and Braenderup	Non-dairy imitation ice cream	11,300 CFU	[22]
NA^2^/150	Typhimurium	Non-dairy imitation ice cream	11,300 CFU	[22]
154/480 (32%)	Typhimurium	Non-dairy imitation ice cream	11,300 CFU	[22]
NA^2^/120	Typhimurium	Non-dairy imitation ice cream	11,300 CFU	[22]
NA^2^/100	NA^2^	Non-dairy imitation ice cream	11,300 CFU	[22]
79/144 (55%)	Typhimurium	Non-dairy imitation ice cream	11,300 CFU	[22]
NA^2^/180	NA^2^	Non-dairy imitation ice cream	11,300 CFU	[22]
47/117 (40%)	Typhimurium var. Copenhagen	Non-dairy imitation ice cream	11,300 CFU	[22]
NA^2^/140	NA^2^	Non-dairy imitation ice cream	11,300 CFU	[22]
NA^2^/150	NA^2^	Non-dairy imitation ice cream	11,300 CFU	[22]
95/NA^2^	Eastbourne	Chocolate balls	99–450 MPN	[23]
80/NA^2^	Eastbourne	Chocolate balls	1125 CFU	[24]
48/NA^2^	Newport	Raw beef patties	<60 to 2300 CFU	[25]
28,000–36,000 /100,000 (estimated) (28%–36%)	Heidelberg	Cheddar cheese	0.1–0.5 CFU	[26]
245/NA^2^	Napoli	Chocolate bars	6 CFU	[27]
245/NA^2^	Napoli	Chocolate bars	25.6 CFU	[28]
8/8 (100%)	Typhimurium	Homemade ice cream	100,000,000–1,000,000,000 CFU	[29]
2/2 (100%)	Typhimurium PT10	Cheddar cheese	4.5 MPN	[18]
2/5 (40%)	Typhimurium PT10	Cheddar cheese	3.6 MPN	[18]
1/2 (50%)	Typhimurium PT10	Cheddar cheese	6.1 MPN	[18]
361/NA^2^	Typhimurium	Chocolate	<10 CFU	[30]
2/3 (66%)	Enteritidis PT4	Vanilla ice cream with chocolate chips	100,000–10,000,000 CFU	[31]
1000/10,000,000 (estimated) (0.01%)	Saint Paul, Javiana, and Rubislaw	Paprika chips and paprika powder	4–45 MPN	[32]
3/3 (100%)	Enteritidis PT8	Ice cream	7–28 MPN	[19]
4/4 (100%)	Enteritidis PT8	Ice cream	25–99 MPN	[19]
1/2 (50%)	Enteritidis PT8	Ice cream	60–270 MPN	[19]
418/2267 (18%)	Enteritidis PT1	Peanut dressing	<8000 CFU	[33]
179/1320 (14%)	Enteritidis	Peanut dressing	344 CFU	[33]
967/10,552 (9%)	Enteritidis PT22	Beef and bean sprouts with sesame dressing	880 CFU	[33]
644/5320 (12%)	Enteritidis PT1	Spinach with peanut dressing	49 CFU	[33]
52/152 (34%)	Enteritidis	Macaroni salad	44,000 CFU	[33]
3/16 (19%)	Enteritidis	Chicken and eggs on rice	4050 CFU	[33]
42/156 (27%)	Enteritidis	Egg salad	31.2 CFU	[33]
36/126 (29%)	Enteritidis PT1	Tartar sauce	<3600 CFU	[33]
45/191 (24%)	Enteritidis	Natto with raw eggs	60,000,000 CFU	[33]
75/343 (22%)	Enteritidis PT4	Grated yam diluted with soup	144,000 CFU	[33]
40/99 (40%)	Typhimurium	Grated yam diluted with soup	138,000 CFU	[33]
39/79 (49%)	Typhimurium	Grated yam diluted with soup (with quail eggs)	2,400,000 CFU	[33]
3/5 (60%)	Enteritidis PT4	Seared beef	240,000 CFU	[33]
558/885 (63%)	Enteritidis PT1	Thin omelette	6000 CFU	[33]
10/11 (91%)	Enteritidis	Omelette	150,000 CFU	[33]
70/104 (67%)	Enteritidis PT1	Broiled tiger prawn with egg yolk	96,000 CFU	[33]
198/363 (55%)	Enteritidis	Plain rolled egg	11 CFU	[33]
30/38 (79%)	Enteritidis	Scallop cream sauce	1,000,000 CFU	[33]
9/9 (100%)	Enteritidis	Natto with raw eggs	720,000 CFU	[33]
113/123 (92%)	Enteritidis	Grated yam diluted with soup	1,900,000 CFU	[33]
73/78 (94%)	Enteritidis	Spaghetti salad	14,000,000 CFU	[33]
57/103 (55%)	Enteritidis	Omelette	240,000 CFU	[33]
100/123 (81%)	Enteritidis	Bavarois	100,000 CFU	[33]
34/68 (50%)	Bareilly O7	Sauce for octopus pancake	14,000,000 CFU	[33]
11/11 (100%)	Oranienburg O7	Grated yam diluted with soup	7,500,000,000 CFU	[33]
5/5 (100%)	Enteritidis	Green tea	3,800,000 CFU	[33]
1,371/5,103 (27%)	Enteritidis PT4	Three-layer cake	420 CFU	[33]
697/7873 (9%)	Enteritidis	Tiramisu	13,000,000 CFU	[33]
11/13 (85%)	Enteritidis	Cake	600,000 CFU	[33]
68/83 (82%)	Enteritidis PT1	Sherbet	<240,000 CFU	[33]
498/2907 (17%)	Enteritidis PT4	Homemade mayonnaise in bread	55,000 CFU	[33]
50/117 (43%)	Enteritidis	Chicken and eggs on rice	4050 CFU	[33]
4/4 (100%)	Enteritidis	Puff cream from a sweet shop	3,510,000 CFU	[10]
19/84 (23%)	Enteritidis	Scrambled egg and soybean cake	400 MPN	[10]
6/6 (100%)	Enteritidis	Cooked rice with red beans from catering meal	2,800,000,000–14,000,000,000 CFU	[10]
75/113 (66%)	Enteritidis	Dumpling with soy sauce and sugar from a sweet shop	81 MPN	[10]
29/59 (49%)	Enteritidis	Rice ball wrapped with deep-fried tofu	72,000,000–360,000,000 CFU	[10]
157/1577 (10%)	Cerro	Bread for hamburger from a bakery	1560 MPN	[10]
12/96 (13%)	Montevideo	Salad with radish sprouts	363 MPN	[10]
17/72 (24%)	Agona	Fried soy pulp and egg	<1500 CFU	[10]
8/9 (89%)	O4 : H : eh,NT	Sushi with fish, egg, and vegetables	259,000 CFU	[10]

^1^ Quantity of *Salmonella* ingested, calculated as the product of the concentration of *Salmonella* in the food and the amount of food consumed. ^2^ Data not available.

#### 3.3.3. Randomised controlled trials

A randomised controlled trial is a type of experimental study designed to assess the impact of an intervention by randomly allocating participants to different groups. The work of McCullough & Eisele, conducted in 1950, represents the only experimental trials identified in our literature search [14]–[17]. These studies involved a population of healthy young adults, specifically volunteers residing in a prison setting. The objective of these trials was to determine the virulence of certain serovars and strains of *Salmonella*. The serovars evaluated included Pullorum (strains I to IV), Newport, Derby, Bareilly, Meleagridis (strains I to III), and Anatum (strains I to III). A total of 199 individuals were included, divided into small groups of five to eight people, with most groups consisting of six individuals. The bacteria were administered individually in varying doses to each group *per os* after the midday meal, suspended in eggnog, a high-fat beverage made from milk, cream, sugar, and egg yolk. To confirm that an exposed individual had developed salmonellosis, the authors established a specific definition. Affected individuals had to display symptoms such as diarrhoea, vomiting, and/or fever, in addition to the presence of *Salmonella* in their stools.

The dose at which at least 50% of the exposed population developed illness for each tested serovar and strain varied from 860,000 for *Salmonella* Anatum I to 10,000,000,000 CFU for *Salmonella* Pullorum I. It is important to note that the infectious doses observed by McCullough & Eisele [14]–[17] were generally much higher than those observed in the previously described studies. The results of the randomised controlled trials are summarised in Table 6.

**Table 6.** Summary of findings of randomised controlled trials.

Population (number of sick/exposed individuals)	Salmonella serovar (and strain)	Tested dose range (CFU)	Dose causing illness in ≥ 50% of the population (CFU)	Source
4/6 (67%)	*Salmonella* Meleagridis I	12,000–50,000,000	50,000,000	[14]
5/6 (83%)	*Salmonella* Meleagridis II	1,000,000–41,000,000	41,000,000	[14]
2/6 (33%)	*Salmonella* Meleagridis III	158,000–10,000,000	>10,000,000	[14]
3/6 (50%)	*Salmonella* Anatum I	12,000–860,000	860,000	[14]
4/8 (50%)	*Salmonella* Anatum II	89,000–67,250,000	67,250,000	[14]
4/6 (67%)	*Salmonella* Anatum III	159,000–4,675,000	4,675,000	[14]
3/6 (50%)	*Salmonella* Newport	152,000–1,350,000	1,350,000	[15]
3/6 (50%)	*Salmonella* Derby	138,000–15,000,000	15,000,000	[15]
4/6 (67%)	*Salmonella* Bareilly	125,000–1,700,000	1,700,000	[15]
6/6 (100%)	*Salmonella* Pullorum I	10,000–16,000,000,000	10,000,000,000	[16]
4/5 (80%)	*Salmonella* Pullorum II	1,387,500–5,750,000,000	5,750,000,000	[16]
6/6 (100%)	*Salmonella* Pullorum III	2,305,000–7,640,000,000	7,640,000,000	[16]
3/6 (50%)	*Salmonella* Pullorum IV	1,880,000–3,975,000,000	1,280,000,000	[16]

#### 3.3.4. Mathematical models

Dose-response modelling is a method used to quantify the relationship between the dose of a pathogen and the probability or severity of an adverse outcome, such as infection, illness, or death, in an exposed population. This approach is crucial in risk assessment as it helps predict the impact of different exposure levels on public health. In this review, dose-response modelling was examined in eight reports and 11 studies.

The first dose-response models for human salmonellosis, published in the late 1990s and early 2000s [34]–[37], were based on data from human feeding studies conducted by McCullough & Eisele [14]–[16]. In 1998, Coleman and Marks noted that data supporting quantitative modelling of dose-response relationships were limited [34]. A key advancement came with the introduction of the distinction between infection and illness in the model by Teunis et al., an essential element in quantitative risk assessment. According to Teunis et al., infection occurs when a pathogen reproduces in the host's digestive tract without necessarily causing symptoms, whereas illness refers to the symptomatic stages, such as acute gastroenteritis and potential chronic complications [35]. Building on this, Latimer et al. introduced an additional variable and developed three dose-response models based on the virulence of various *Salmonella* serovars and strains [36]. They categorised *Salmonella* Anatum II and *Salmonella* Meleagridis I as low virulence strains, causing disease at high doses, while *S*. Anatum I, *Salmonella* Bareilly, and *S*. Newport were modelled as causing disease at moderate doses. Since human feeding study data for highly virulent *Salmonella* strains were unavailable, *Shigella dysenteriae* was used as a proxy, drawing on Levine et al.'s results [38]. This was the only study to use another bacterium to model *Salmonella* effects. Finally, Oscar's 2004 study was the last, as retrieved in this review, to use McCullough & Eisele's dataset [14]–[16] as the sole data source. Oscar developed a three-phase linear model to determine the minimum, median, and maximum illness doses for 13 *Salmonella* strains [37]. In this study, the lowest minimum illness dose observed was 5.9 organisms for *S*. Anatum I, while the highest was 9.7 organisms for *S*. Pullorum I.

The other four retrieved records [39]–[42] included studies that integrated outbreak data, allowing for the consideration of real-life conditions such as variations in the food matrix or susceptibility to illness. However, these datasets are subject to greater uncertainties compared to clinical trials. The WHO published a mathematical model in 2002 based on outbreak data obtained from published literature, national reports, and unpublished data [39]. This model did not provide evidence that the dose of *S*. Enteritidis causing illness was different from other serovars, nor did it suggest an increased risk of illness in children under five years compared with the rest of the population exposed to *Salmonella*. However, it is recognised that the database may lack sufficient power to reveal true differences that might exist. Based on the same WHO dataset [39], Bollaerts et al. estimated the dose-illness relationship for *Salmonella*. Their main findings included the influence of the serovar × food combination on the probability of illness and the existence of immunity in the general population but not in the vulnerable or at-risk population [40]. Teunis et al. included data from both human feeding studies and outbreaks in their mathematical model. The model also distinguished between infection and illness, estimating an infection ID50 of 7 CFU and an illness ID50 of 36 CFU [41]. Lastly, Akil & Ahmad developed a quantitative risk assessment model for human salmonellosis resulting from the consumption of broiler chicken. For their dose-response model, they used data retrieved by the WHO [39] and Teunis et al. [41], determining the ID50 for two *Salmonella* serovars: 1.5 × 10^4^ organisms for *S*. Enteritidis and 6.4 × 10^3^ organisms for *S*. Typhimurium [42].

## 4. Discussion

Salmonellosis is a major zoonotic disease worldwide, and non-typhoidal *Salmonella* is well-represented in foodborne illness episodes. Understanding its virulence and the factors that influence it is crucial for improving public health responses to this threat. In this review, we aimed to investigate the relationship between the dose of non-typhoidal *Salmonella* and foodborne illness in humans.

We observed significant variability in the types of studies and methodologies, including differences in exposed populations, *Salmonella* serovars and strains, vector foods, and study conditions. Despite these variations, several key observations emerged.

During our literature search, we identified several studies that reported *Salmonella* levels in food products linked to outbreaks. These studies provide valuable data on contamination levels in different food matrices; however, they often lack information on the quantity of food consumed by affected individuals [43],[44]. Since calculating an accurate exposure dose requires both *Salmonella* concentration and the quantity of food ingested, studies without ingestion data or reasonable estimates could not be included in our systematic review.

The most frequently studied and represented serovars in the literature were Enteritidis and Typhimurium. The serovar (and strain) of *Salmonella* is a critical factor to consider in any type of study because the virulence of *Salmonella* varies significantly between serovars. For example, *S*. Enteritidis and *S*. Typhimurium are among the most common causes of human salmonellosis worldwide due to their ability to persist in various food products and effectively colonise the human gastrointestinal tract [45],[46]. In contrast, other serovars, such as *S*. Agona or *S*. Montevideo, are less frequently implicated in human infections, possibly due to differences in host adaptation and transmission dynamics [47]. The ability of certain serovars to survive in specific food matrices and resist environmental stressors further influences their role in foodborne outbreaks [48]. Given these variations, serovar identification is essential for understanding the risk posed by different *Salmonella* strains and for developing targeted control strategies. Therefore, an accurate comparison of two doses requires them to involve the same bacterium and hence the same serovar. This need for consistency in serovars has posed a significant challenge in conducting comparative studies of foodborne illness outbreaks documented in case series. Additionally, even when the same serovar is involved, comparisons may still be difficult, as bacteria evolve over time, leading to the emergence of different strains. This evolving nature of bacteria makes it particularly difficult to evaluate virulence in field studies. Moreover, laboratory methods primarily focus on identifying serovars based on the distinct combination of O and H antigens, but they do not routinely assess virulence (e.g., molecular detection of virulence genes or cell invasion studies). Consequently, the same serovars may be identified, yet their infectious properties can vary, particularly with changes over time.

A wide variety of foods were implicated in the studies reviewed, with eggs and egg products frequently reported as sources of salmonellosis in outbreak investigations. The composition of the food plays a crucial role in understanding the severity of salmonellosis, and there are several precautions to consider when interpreting studies on foodborne transmission. For instance, *Salmonella* can become trapped in lipid-rich products, enabling it to survive the acidic conditions of the stomach and subsequently proliferate in the intestinal tract [18]. Therefore, conclusions regarding the infectious dose can only be made accurately when the specific characteristics of the food are well understood, making it essential to be as precise as possible about the contaminated dish or meal leading to salmonellosis. Additionally, providing the physicochemical properties of the food, such as pH, water activity (*a*_w_), and protein and fat content, would enhance the ability to compare different foods in terms of their potential to harbour *Salmonella* and cause illness.

An additional issue concerning food is the uneven distribution of bacteria within it. In experimental studies, liquid food is often used, allowing for a more uniform concentration of *Salmonella*. However, in the outbreaks included in our review, liquid foods were less frequently represented. Typically, entire dishes become contaminated, and the bacterial load is probably not evenly distributed among the various components of the meal. This uneven distribution means that some individuals may be exposed to significantly higher levels of *Salmonella* than others. While the *Salmonella* hazard affects the entire population exposed, the actual risk of illness depends on the level of exposure for each individual. Assuming a uniform bacterial concentration and consistent consumption can lead to inaccurate estimations of the infectious dose. To better assess the risk posed by contaminated food, a more precise analysis of samples from different parts of the affected dish is necessary. This would provide a clearer understanding of the bacterial load and help determine the quantity needed to cause illness more accurately. Another crucial factor to consider is the sample storage conditions before analysis, as they can significantly influence the quantitative results of laboratory tests. Once the initial cases of salmonellosis are identified, an investigation is often required to trace the source of the outbreak. During this period, the food may be stored under conditions that either promote the multiplication or destruction of *Salmonella*. Consequently, the concentration of *Salmonella* detected in the analysis may differ from the concentration that initially caused the foodborne illness. Studies by Armstrong et al., Fontaine et al., D'Aoust, Morgan et al., Lehmacher et al., and Vought & Tatini accounted for this factor in their dose calculations [18],[19],[22],[25],[31],[32]. In contrast, other authors either acknowledged that storage conditions may influence the results but did not incorporate this into their dose calculations or omitted any mention of storage conditions altogether.

The lowest dose of *Salmonella* that caused illness in at least one individual was 0.7 MPN [18], while doses as high as 14,000,000,000 organisms were also reported [10]. Lower doses of *Salmonella* were observed in cases involving vulnerable populations, such as children and the elderly, as well as in instances where contaminated products reached a large number of people simultaneously. Several factors influence the likelihood of illness after exposure to *Salmonella*, with an individual's health status being a key determinant of their susceptibility to salmonellosis. For instance, case reports indicated that the ingested dose required to cause illness in children and elderly individuals was significantly lower compared to studies focusing on the general population [18],[21]. While the health status of exposed individuals was typically well documented in case reports, such information was often lacking in case series. For example, the case series by Hara-Kudo & Takatori, Armstrong et al., Fontaine et al., Greenwood & Hooper, and Morgan et al. provided limited or no details on the health status of the populations studied [10],[22],[25],[28],[31]. In some cases, this limitation may be attributed to privacy concerns and ethical restrictions, which make it challenging for researchers to collect and disclose personal health information in retrospective outbreak investigations. In contrast, the health status of individuals was well documented in the studies conducted by McCullough & Eisele [14]–[16], which involved only healthy young male volunteers, some of whom were exposed to multiple doses of *Salmonella*, potentially contributing to their immunity before becoming ill. However, individual characteristics such as sex, age, underlying health conditions, and immune status vary, making the general population more susceptible overall. As a result, the doses causing infection and illness in controlled studies were inevitably higher than those that would trigger the same effects in the broader population.

Another important factor for understanding the dose-response relationship of pathogenic bacteria is the attack rate, which is calculated based on the number of exposed and ill individuals. For example, in the case series reported by Fontaine et al. and Lehmacher et al., the attack rate was successfully estimated [25],[32]. By contrast, in the studies by Armstrong et al., Craven et al., D'Aoust et al., Fontaine et al., Greenwood & Hooper, Gill et al., and Kapperud, the available data were insufficient to determine an accurate attack rate [22]–[25],[27],[28],[30]. The ingested dose is another significant consideration. In most studies, the reported dose was an estimate of *Salmonella*. However, in 19 studies from the records by Kasuga et al. and Hara-Kudo & Takatori, the dose was precisely measured [10],[33]. Finally, in the models developed by Teunis et al. and Akil et al., the method for establishing the dose-response relationship was not clearly explained [41],[42].

To summarise, early experimental studies, which cannot be reproduced today, provided valuable control over certain parameters that influence the infectious dose, such as the implicated food, the precise ingested dose, and the health status of the exposed population. However, the fixed nature of these parameters resulted in infectious doses that were often artificially high and far removed from real-world infection conditions. In contrast, field observations have become a crucial source of information through the analysis of foodborne outbreaks and clinical cases. These real-world data offer insights into a wide range of *Salmonella* serovars, various contaminated foods, and a diverse population with individuals exhibiting unique characteristics that influence their susceptibility to infection. Nevertheless, this variability complicates the comparison of studies. Additionally, since the information is often collected retrospectively, it tends to be incomplete, and necessary approximations can hinder the accuracy of the infectious doses reported. It is important to acknowledge that a significant number of salmonellosis cases occur sporadically and are not linked to any specific outbreak. These cases are often associated with the consumption of raw or undercooked meat products—such as poultry, pork, and beef—as well as vegetables [49],[50]. The underrepresentation of such sporadic cases in the scientific literature may lead to an incomplete understanding of the true burden of salmonellosis across different food matrices. To address the lack of comprehensive data, mathematical modelling has become an important tool. This approach allows for the use of incomplete data from outbreaks, clinical cases, or experimental trials to extrapolate and create dose-response models that assess the risk of infection or illness under specific conditions. Although this type of study remains reliable for understanding the dose-response relationship, the persistent lack of real-world data continues to present a significant limitation.

## 5. Conclusions

In conclusion, efforts must be dedicated to the detection and investigation of *Salmonella* outbreaks to gather sufficient high-quality data to improve dose-response models. The low number of studies selected for this review confirms the scarcity of such data, as outbreak investigations do not always result in the determination of the pathogen dose that causes illness. Gaining a better understanding of this relationship will allow for the development of more targeted research and control measures, ultimately strengthening the protection of populations against the foodborne risk posed by *Salmonella*. This study provides a comprehensive compilation of all published data on the relationship between non-typhoidal *Salmonella* doses and foodborne infections in humans. These findings can serve as a valuable resource for future mathematical modelling efforts, contributing to ongoing efforts to enhance food safety.

## Use of AI tools declaration

The authors declare they have not used Artificial Intelligence (AI) tools in the creation of this article.
